# Impact of Focal Muscle Vibration on Flaccid Upper Limb Motor Paralysis following Acute Brain Disease: A Case Study

**DOI:** 10.1155/2024/2469074

**Published:** 2024-06-25

**Authors:** Hirotaka Saito, Haruka Kobayashi, Kodai Oba, Yosuke Hamaya

**Affiliations:** ^1^ Department of Rehabilitation Medicine St. Marianna University School of Medicine Hospital, Kawasaki, Japan; ^2^ Department of Rehabilitation Medicine Dokkyo Medical University Saitama Medical Center, Koshigaya, Japan

## Abstract

Focal muscle vibration (FMV) is increasingly being recognized as a rehabilitative therapy for enhancing motor function in central nervous system (CNS) diseases, particularly in patients with fine motor control deficits stemming from CNS damage. Brain lesions from these diseases disrupt the motor networks, necessitating novel rehabilitation strategies. By applying vibrations to muscles, FMV stimulates sensory fibers to induce cortical activity and kinesthetic illusions. While initial studies have highlighted FMV's role in reducing spasticity, recent evidence points to its potential in treating motor paralysis. However, prior research has been limited by the lack of acute-phase studies and a focus on patients with minimal muscle contraction capability. This report aimed to explore FMV's efficacy on upper limb motor function in patients with flaccid motor paralysis immediately after acute CNS diseases. We report the case of a septuagenarian male with a brain abscess in the right parietal lobe, leading to flaccid motor paralysis. Rehabilitation included 28 sessions of occupational and physical therapy that incorporated FMV. Significant improvements were observed in upper extremity function, with moderate to very large effect sizes, while lower limb function showed lesser improvement without adverse effects. This case suggests the utility of FMV in enhancing upper-limb motor function after acute CNS injuries, potentially serving as a supplementary therapy for spontaneous recovery. This report contributes to emerging evidence on FMV's benefits in acute flaccid motor paralysis, expanding the documented therapeutic scope.

## 1. Introduction

In recent years, focal muscle vibration (FMV) has emerged as a noteworthy therapeutic intervention for enhancing motor function in central nervous system (CNS) diseases [1–4]. The impairment of fine motor control, which relies on proprioceptive feedback, can be particularly pronounced in individuals with post-CNS disease, leading to substantial deficits in upper-limb motor control [5–7]. Indeed, robot-assisted sensorimotor training has demonstrated efficacy in ameliorating proprioception and upper limb motor function in stroke patients [8–11]. Focal brain lesions resulting from CNS diseases give rise to both structural and functional alterations in the vicinity of the lesion and in distant regions [12–15]. Focal brain lesions may disrupt motor descending tracts by introducing imbalances in excitatory and inhibitory processes within pertinent regions of the motor network, affecting both the affected and unaffected hemispheres. Therefore, modulation of motor networks, fostering brain plasticity and reorganization, is imperative for motor function rehabilitation following stroke and other CNS diseases.

FMV entails repetitive application of proprioceptive stimuli via a mechanical device [1–4, 16]. Physiologically, vibrations applied to muscles or tendons trigger the firing of Ia afferent sensory fibers, subsequently manifesting as cortical activity [16–19]. Activation of Ia [20] and II [21] afferent sensory fibers through muscle spindles generates a sensation of limb movement, even in the absence of voluntary motion. Previous investigations have reported comprehensive brain activity patterns correlated with the motor network during FMV-induced kinesthetic illusion [22–25]. Importantly, this kinesthetic illusion does not solely result from corollary discharges from the motor cortex to the sensory cortex, which co-occur with motor commands, but is primarily induced by signals from afferent sensory fibers in response to FMV [26]. This underscores the significance of the kinesthetic illusion elicited by FMV as a modulator of the motor network in addressing motor impairments following CNS disease.

Most studies supporting FMV's efficacy in mitigating motor impairments post-CNS disease have focused on spasticity. In recent years, a growing body of research has reported favorable outcomes of FMV on motor paralysis [27–29]. However, most of these previous investigations were conducted during the chronic phase [1–4, 27, 28], with only limited validation in the acute phase, as reported by Toscano et al. [29]. Furthermore, in most cases, the targeted level of motor paralysis necessitated muscle strength above the minimal isometric voluntary contraction [1–4, 27–29]. Epidemiological studies have indicated a decline in stroke-related mortality, the most prevalent CNS disease, while the number of survivors is rising [30]. Although Toscano et al. [29] demonstrated the beneficial effects of FMV on upper limb motor paralysis in acute stroke patients, it is crucial to note that the subjects possessed muscle strength above the minimal isometric voluntary contraction threshold. In essence, there is a dearth of reports examining the impact of FMV on flaccid upper limb motor paralysis immediately following acute CNS diseases.

FMV is clinically significant because of its ability to be safely and efficiently administered at the bedside in acute medical settings that require close supervision [31]. Furthermore, this report pioneered an investigation into FMV's impact on flaccid motor paralysis of the upper limbs in the acute phase of post-CNS diseases, a demographic previously underrepresented in FMV research. Therefore, the primary objective of this report was to investigate FMV's effects on upper limb motor function in a patient with flaccid motor paralysis immediately after acute brain disease.

## 2. Case Presentation

### 2.1. Participant

**Figure 1:**
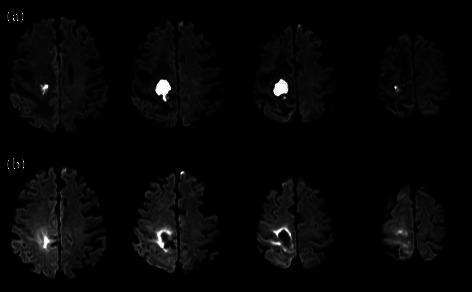
Diffusion-weighted imaging: transaxial slices of the brain. (a) Images obtained one day post-onset demonstrate a cortical lesion located in the right parietal lobe, predominantly affecting the right cerebral hemisphere. The lesion, characterized as an abscess, measured up to a maximum diameter of 21 mm. (b) Subsequent imaging conducted 43 days after the initial presentation showing a decrease in lesion size within the right parietal lobe, with the maximum diameter reduced to 14 mm.

The subject of this report was a right-handed male in his seventies with a documented history of hypertension. Diffusion-weighted imaging revealed that the patient was afflicted with a brain abscess measuring up to 21 mm in maximum diameter located within the right parietal lobe (Figure 1). Immediate antimicrobial therapy was initiated upon detection of the condition. Subsequently, a comprehensive rehabilitation program encompassing occupational and physical therapy was initiated on the second day following the diagnosis of brain abscess. On the 41st day of hospitalization, the patient was transferred to a rehabilitation facility.

The patient exhibited neurological symptoms characterized by atonic muscles in the left upper and lower extremities, as indicated by a Modified Ashworth Scale score of 0/4. On assessment using the NIH Stroke Scale, the patient scored 8, with both motor arm and leg functions rated 4/4. Remarkably, the patient maintained clear consciousness, and no sensory deficits, aphasia, or hemispatial neglect were evident. However, due to flaccid motor paralysis, the patient's mobility was severely restricted, necessitating assistance in activities of daily living. Cognitive assessment scores, as measured by the Mini-Mental State Examination-Japanese [32] and the Montreal Cognitive Assessment Japanese [33], were both recorded out of 23/30 (Table 1).

The objective of this report was elucidated to the patient in compliance with international legal regulations, as stipulated in the Declaration of Helsinki from 1964. It is important to note that case reports are exempt from review by our Ethics Committee. The patient involved in this study provided written informed consent for publication.

### 2.2. Study Design

**Figure 2:**
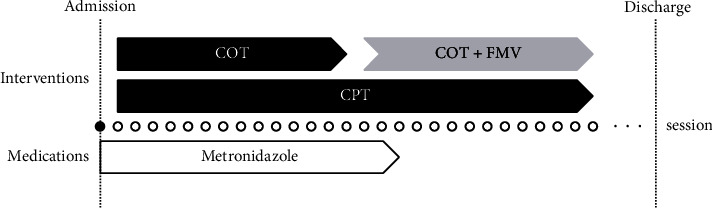
Overview of intervention procedures. COT: conventional occupational therapy, CPT: conventional physical therapy, and FMV: focal muscle vibration.

This report had a prospective, single-case experimental design. The rehabilitation therapy regimen encompassed 28 sessions, comprising 14 sessions each for the baseline and intervention periods (Figure 2). Each session lasted 80 min, with 40 min dedicated to occupational and physical therapy. In total, 2,240 min of rehabilitation were administered across these 28 sessions. A minimum one-hour rest interval was observed between the occupational therapy and physiotherapy training sessions.

For upper limb motor paralysis, the baseline period exclusively involved conventional occupational therapy, whereas the intervention period included FMV in conjunction with conventional occupational therapy. Conversely, lower limb motor paralysis patients received only conventional physical therapy, consisting of sit-to-stand and standing exercises, in both report phases.

Statistical analysis was conducted using the Brunner-Munzel test to evaluate the relationship between baseline and intervention periods. Nevertheless, both upper- and lower-limb motor functions exhibited significant changes during the intervention period, making it difficult to attribute these effects solely to FMV. Therefore, effect sizes were calculated using the tau-*U* test [34], and an in-depth analysis of the impact of FMV on motor function was conducted. R was used to compare the baseline and intervention periods [35], and the effect sizes for upper and lower limb motor function were calculated using an online web-based calculator [36].

### 2.3. Outcome Measures

The evaluation of motor function in both upper and lower extremities was conducted using standardized assessment scales, including the NIH Stroke Scale motor items [37] and Motricity Index [38]. In addition, upper extremity motor function was further evaluated using the Fugl-Meyer Motor Function Assessment-Upper Extremity (FMA-UE) [39]. Grip strength was measured using a hand dynamometer (Jamar, JLW Instruments Co., Ltd., IL, US) [40]. The physical therapist measured the lower limb motor function, and the occupational therapist measured the upper limb motor function after each session. The evaluations were performed 14 times each during the baseline and intervention periods for a total of 28 evaluations.

### 2.4. Treatment

#### 2.4.1. Conventional Therapy

Patients underwent daily rehabilitation sessions lasting 80 min each. These sessions included 20–40 minutes of conventional occupational therapy and 40 minutes of conventional physical therapy. Conventional occupational therapy primarily focuses on bilateral upper-extremity training, as described in previous studies [41, 42]. Conventional physical therapy comprises high-intensity exercises consistent with established protocols [43, 44].

During bilateral upper-extremity training, the therapist supported the paralyzed patient's arms to the extent that the patient could self-support. This regimen included five sets, each comprising 20 repetitions, with brief intervals between the sets. However, it is pertinent to mention that bilateral upper extremity training was reduced to 20 minutes during the intervention period due to the incorporation of FMV.

High-intensity training encompassed repetitive activities, such as sit-to-stand, stand-to-sit, and gait training, with the therapist offering appropriate support to the patient throughout the exercises.

#### 2.4.2. Focal Muscle Vibration (FMV)

Two occupational therapists administered FMV using a household hand massager (THRIVE MD-01; Thrive Co., Ltd., Osaka, Japan) exclusively during the intervention period. Based on previous studies, the specific target muscles were the biceps brachii, triceps brachii, flexor carpi radialis, and extensor carpi radialis muscles [1, 3, 4]. Vibration stimulation was intermittently applied to the distal tendon of each target muscle for 5 min, with 1-minute breaks, while the patient assumed a supine position on the treatment bed.

The selected stimulation frequency of 91.7 Hz was consistent with previous findings, as FMV reportedly induces kinesthetic illusions within the range of vibration frequencies from 80 Hz to 100 Hz [22–25]. The amplitude was standardized to 2 mm. Patients were instructed to keep their eyes closed during FMV sessions, as the visual perception of a fixed limb has been shown to attenuate the kinesthetic illusion [45]. Furthermore, considering the potential impact of motor imagery on cortical activity and connectivity [46], patients were encouraged to mentally envision joint movements that opposed the action of the target muscle during the vibratory stimulus.

## 3. Results

**Figure 3:**
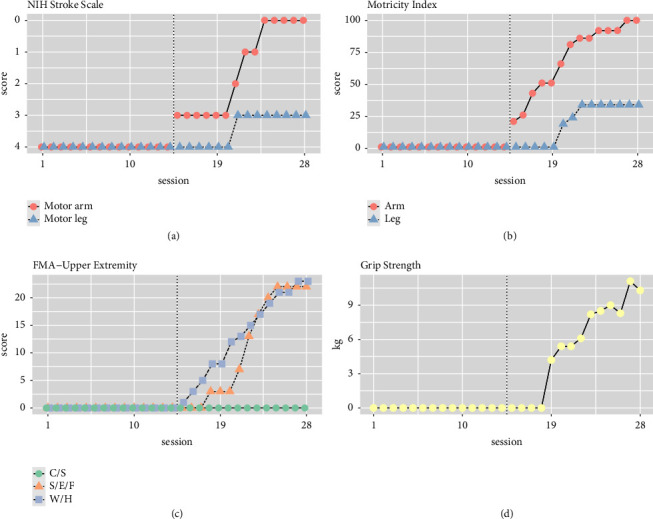
Comparative assessment of upper- and lower-extremity motor functions across the two periods. (a) Changes in the National Institute of Health Stroke Scale (NIHSS). (b) Changes in the Motricity Index for Motor Function Assessment. (c) Changes in the Fugl-Meyer Assessment for Upper Extremity (FMA-UE), which evaluates post-stroke motor recovery. (d) Changes in grip strength as a measure of hand function. S/E/F: shoulder/elbow/forearm, indicating the segments of the upper extremity evaluated. W/H: wrist/hand, indicating the segments of the distal upper extremity evaluated. C/S: coordination/speed, aspects of motor function assessed in the evaluations.

The results of the Brunner-Munzel test showed significant changes in both upper limb motor function and lower limb motor function during the intervention period (Figure 3). In other words, it was not possible to compare the degree of improvement in upper limb and lower limb motor function during the intervention period.

The NIH Stroke Scale and Motricity Index scores for the paralyzed upper extremity demonstrated substantial improvements, transitioning from 4 to 0 and from 1 to 100, respectively (Figure 3). The calculated effect sizes (tau) for the NIH Stroke Scale were −1 (indicating a very large change) for the upper extremity and −0.5714 (indicating a moderate change) for the lower extremity. Similarly, the total Motricity Index showed effect sizes of 1 (indicating a very large change) for the upper extremity and 0.6429 (indicating a moderate change) for the lower extremity. Further breakdown of the Motricity Index into subscales revealed tau values of 0.8571 (indicating a very large change) for the shoulder, 1 for the elbow, and 1 for the finger in the upper extremity subscale. In contrast, the lower extremity subscale exhibited tau values of 0.6429 (indicating a large change) for the hip, 0.6429 (indicating a large change) for the knee, and 0 (indicating no change) for the ankle. Notably, the effect size was more pronounced for the upper limb than for the lower limb in both the NIH Stroke Scale and Motricity Index motor function scales (Figure 3).

The Fugl-Meyer Motor Function Assessment-Upper Extremity (FMA-UE) total score, its subscales (except for coordination speed), and grip strength results are presented in Table 2. The tau values for FMA-UE were 1 (indicating a very large change) for the total score, 0.7857 (indicating a large change) for the shoulder-elbow-forearm, 1 (indicating a very large change) for the wrist and hand, and 0 (indicating no change) for coordination speed. Grip strength displayed a tau value of 0.7143 (indicating a large change). Improvement in upper limb motor function was observed in the joints targeted by FMV (finger and elbow) and in those not directly involved (shoulder).

During the entire intervention period, spanning all 14 sessions, no adverse effects or complaints were reported, either during or after the application of FMV.

## 4. Discussion

The present report demonstrated the effectiveness of FMV in enhancing upper limb motor function in a patient with flaccid motor paralysis following acute brain injury due to a brain abscess in the right parietal lobe region. This research marks a pioneering effort as the first report to investigate the effects of FMV on flaccid upper limb motor paralysis after acute CNS disease.

The observed improvements in the NIH Stroke Scale and Motricity Index scores tended to be more pronounced for the upper extremities than for the lower extremities following the initiation of FMV treatment. However, this outcome requires careful consideration of the potential influence on spontaneous recovery. Physiological processes drive brain changes resulting from spontaneous recovery and can be categorized into three recovery epochs [47]. The initial epoch of spontaneous recovery occurs within the first few hours of disease onset and is characterized by reperfusion and neuroprotection to salvage at-risk tissues. The second epoch unfolds during the initial days to weeks after disease onset, marking a phase of heightened spontaneous recovery during which the most significant improvements typically occur. The third epoch signifies the chronic phase of brain repair, during which endogenous brain repair mechanisms become relatively stable, yet brain structural and functional changes can still manifest. The observed improvements in upper and lower limb motor function within two weeks suggest that spontaneous recovery significantly contributed to the enhancement of the patient's motor function.

Of particular interest, the patient exhibited better recovery of motor function in the upper extremity when FMV was applied compared to the lower extremity. Clinical experience often highlights that motor paralysis in CNS diseases tends to be more severe in the upper extremities and is associated with a less favorable prognosis [48–51]. For instance, Kwakkel et al. [50] reported that following a 16-week follow-up of motor paralysis in acute stroke cases, motor function scores for the lower extremity (FMA-lower extremity and Motricity Index-leg) improved by more than 50%. In contrast, motor function scores for the upper extremity (FMA-UE and Motricity Index-arm) did not reach half of their initial values [50]. This discrepancy can be attributed to the intricate and lateralized function of the corticospinal tract, which has limited compensatory capacity for fine finger movements and dexterity [52].

Moreover, most corticospinal tracts involved in motor output originate from the primary motor cortex, with contributions from multiple cortical regions [53]. Despite the patient's focal lesion centered in the right parietal lobe, the presence of flaccid motor palsy underscores the impact of brain lesions on relevant regions within the motor network in both the affected and unaffected hemispheres, a phenomenon supported by several studies [12–15]. The discrepancy between our findings and previous studies [48–51] may suggest that FMV complements spontaneous recovery.

There is limited empirical evidence concerning the use of FMV for motor paralysis resulting from acute CNS diseases. In [29], the FMV and control groups were compared and validated using outcomes similar to those employed in our report. Their results demonstrated significant improvements in the group receiving FMV, with notable changes observed in the NIH Stroke Scale, Motricity Index, and Fugl-Meyer Assessment (FMA) scores, all of which are established measures of motor function. While our findings align with theirs, it is worth noting that Toscano et al.'s study [29] included patients with muscle strength above the minimal isometric voluntary contraction threshold. Additionally, our report differs from theirs in several methodological aspects. Toscano et al. [29] administered FMV at a frequency of 100 Hz with an amplitude ranging from 0.2 to 0.5 mm on the target muscle, utilizing three sets of 10-minute exposures, each separated by one-minute intervals.

In contrast, our report employed shorter exposure duration and a greater amplitude. Previous research studies exploring the effects of FMV on motor impairment following CNS diseases [1–4] and its relationship with cortical activity [16, 17] have suggested that the effects of FMV may be dose-dependent. Nevertheless, it is essential to acknowledge that none of these studies can be directly extrapolated to our patient, as they primarily focused on patients with chronic stroke patients [1] and healthy subjects [16, 17]. Consequently, determining the optimal duration of FMV exposure for motor paralysis following acute CNS disease remains a challenge. Despite some methodological differences from previous studies, this report provides novel and valuable insights by demonstrating the efficacy of FMV in patients who do not even have minimal isometric voluntary contraction muscle strength.

## 5. Conclusion

The findings presented in this report underscore the promising role of FMV therapy in the rehabilitation of upper limb motor function in patients with flaccid motor paralysis following acute brain disease. The significant improvements observed in the motor functions of the upper extremity, as compared to the lower extremity, in a patient with a brain abscess highlight the potential of FMV to complement traditional rehabilitation efforts and spontaneous recovery processes. This case report not only extends the existing body of literature by validating the efficacy of FMV in the acute phase of brain injury—a period previously underexplored—but also illuminates the importance of including patients with minimal to no muscle strength in FMV research. While the observed benefits are encouraging, they also underscore the necessity for further research to unravel the mechanisms behind FMV's effectiveness, identify optimal treatment parameters, and investigate the relationship between the site of injury and therapeutic outcomes. Moreover, expanding the study to include a larger sample size is critical for generalizing these findings. In conclusion, FMV represents a viable and safe adjunctive therapy for enhancing motor recovery in the acute phase of CNS diseases, potentially setting a new direction for rehabilitation protocols in such patients.

## Figures and Tables

**Table 1:** Patient's characteristics.

	*N* = 1
Age (years)	77
Gender	Male
Diagnosis	Abscess
Time after onset (day)	2
Lesion area	Parietal lobe (max. 21 mm)
Modified Ashworth Scale	
Muscle tone	0 (no increase in muscle tone)
NIH Stroke Scale	
LOC	0 (alert)
LOC questions	0 (answers both correctly)
LOC commands	0 (obeys both correctly)
Motor arm (affected side)	4 (no movement)
Motor leg (affected side)	4 (no movement)
Sensory	0 (normal)
Best language	0 (no aphasia)
Extinction and inattention	0 (no neglect)

LOC: level of consciousness.

**Table 2:** Period changes in upper and lower extremity outcomes.

	Baseline to intervention					
	Brunner-Munzel (*P* value)	Tau	*P* value	90% CI		Effect size
Upper extremity						
NIH Stroke Scale-motor	<0.05	−1	0	−1	−0.635	Very large change^*∗∗∗*^
Motricity Index-total	<0.05	1	0	0.619	1	Very large change^*∗∗∗*^
Shoulder	<0.05	0.8571	0.0001	0.492	1	Very large change^*∗∗∗*^
Elbow	<0.05	1	0	0.635	1	Very large change^*∗∗∗*^
Finger	<0.05	1	0	0.635	1	Very large change^*∗∗∗*^
FMA-UE-total	<0.05	1	0	0.635	1	Very large change^*∗∗∗*^
Shoulder-elbow-forearm	<0.05	0.7857	0.0004	0.42	1	Large change^*∗∗*^
Wrist-hand	<0.05	1	0	0.635	1	Very large change^*∗∗∗*^
Coordination-speed	NA	0	1	−0.365	0.365	No change
Grip	<0.05	0.7143	0.0013	0.349	1	Large change^*∗∗*^
Lower extremity						
NIH Stroke Scale-motor	<0.05	−0.5714	0.0101	−0.937	−0.206	Moderate change^*∗*^
Motricity Index-total	<0.05	0.6429	0.0038	0.278	1	Large change^*∗∗*^
Hip	<0.05	0.6429	0.0038	0.278	1	Large change^*∗∗*^
Knee	<0.05	0.6429	0.0038	0.278	1	Large change^*∗∗*^
Ankle	NA	0	1	−0.365	0.365	No change

Small change: tau < 0.20, ^*∗*^0.20 ≦ tau < 0.60, ^*∗∗*^0.60 ≦ tau < 0.80, and ^*∗∗∗*^tau ≧ 0.80. FMA-UE: Fugl-Meyer Motor Function Assessment-Upper Extremity. NA: not available.

## Data Availability

The clinical data used to support the results of this report are included within the article.
